# Optimizing Patient Risk Stratification for Colonoscopy Screening and Surveillance of Colorectal Cancer: The Role for Linked Data

**DOI:** 10.3389/fpubh.2017.00234

**Published:** 2017-09-08

**Authors:** David B. Preen, Iris Lansdorp-Vogelaar, Hooi C. Ee, Cameron Platell, Dayna R. Cenin, Lakkhina Troeung, Max Bulsara, Peter O’Leary

**Affiliations:** ^1^Centre for Health Services Research, School of Population and Global Health, The University of Western Australia, Perth, WA, Australia; ^2^Department of Public Health, Erasmus University Medical Centre, Rotterdam, Netherlands; ^3^Department of Gastroenterology, Sir Charles Gairdner Hospital, Nedlands, WA, Australia; ^4^Colorectal Cancer Research Unit, The University of Western Australia, Perth, WA, Australia; ^5^Institute for Health Research, University of Notre Dame, Fremantle, WA, Australia; ^6^Faculty of Health Sciences, Curtin University, Perth, WA, Australia

**Keywords:** colorectal cancer, colonoscopy, screening, clinical guidelines, adenoma, risk stratification

## Introduction

Colorectal cancer (CRC) is the third most common cancer worldwide, with an estimated 1.4 million new cases and almost 700,000 related deaths globally each year (1). In Australia, CRC is the second most commonly reported cancer and second most common cause of cancer-related death (2). Moreover, Australia has the fourth highest incidence of CRC for men and fifth highest for women internationally (3, 4). Incidence rates of CRC have at least doubled in many countries since the mid-1970s (5–7), although trends vary across countries with stabilizing or declining rates in more recent years reported in Western Europe and the United States (US), respectively. This trend is reversed for high-income nations that have recently made the transition from low-income economies (8, 9).

In the majority of cases, CRC develops from non-malignant precursor adenomatous colonic polyps (adenomas) (10), with the overall adenoma burden dependent on the number, size, villosity, dysplasia grade, and location of adenomas in the colon. Importantly, the average interval from adenoma appearance to development of CRC is >10 years (11), and the removal of adenomas reduces CRC incidence and mortality (12, 13). This affords an excellent opportunity for early detection through screening and regular colonoscopic surveillance, and the condition meets the World Health Organization criteria for diseases suited to screening (14). Patients with prior adenoma are therefore recommended to undergo regular surveillance colonoscopy (15). Increased surveillance, in addition to advances in surgical and adjuvant therapy (16), has been shown to reduce CRC incidence and increase median 5-year survival for CRC from 55.0% in the early 1980s to 65.3% by 2005 (16).

Lifetime prevalence of adenoma is 40–50% (17), however, the majority of adenomas never develop into malignant neoplasms and only 4–5% of the population eventually develop CRC (18). Consequently, simply identifying the presence of adenomas does not represent the most efficient approach for making informed recommendations for the need and timing of follow-up colonoscopic surveillance and the overall adenoma burden and specific adenoma characteristics should be factored into clinical decision making (12, 13).

## Use of Colonoscopy for CRC Detection

Although some population-based screening programs exist employing fecal occult blood testing (FOBT), colonoscopy remains the “gold-standard” for detection of CRC and precursor adenomas (19). However, others have suggested that colonoscopy is overused as a primary screening and surveillance tool leading to sizable increases in the rates of colonoscopy in many countries (20–22).

In Australia, rising usage of colonoscopy has been seen for over two decades, with Medicare claims for the procedure increasing by 250% in the last 10 years (23). This increase has occurred simultaneously with increased capacity within the private hospital sector (24). Given the current trajectory, and when considered with population aging and the promotion of earlier screening, it is estimated that over 1 million colonoscopies will be performed annually by 2020 in Australia (population 24 million) (25). Similar relative trends have been reported elsewhere, with greater absolute increases, in countries such as the US (26). Such demand is not sustainable for most health systems, both in terms of provider capacity and health-care costs, estimated to be in the multiple billions of dollars annually in western nations (27). Furthermore, if projected increases in demand are realized, access to this service will be compromised, especially in public health systems. Already in Australia waiting times for colonoscopy exceeding 250 days are not uncommon (28, 29).

## Risk Stratification Approaches to CRC Detection and Prevention

Researchers, including our team, have previously called for greater consideration of personalized risk stratification approaches to primary screening for CRC (30); however, less consideration has been given to the potential benefits of such approaches for ongoing surveillance. Targeting colonoscopy to patients who stand to benefit most (i.e., those at higher risk of CRC) through robust risk stratification would reduce the burden of colonoscopies to both patients and the health system, while maintaining the preventive benefits of surveillance colonoscopy. Such targeting could reduce burden for lower-risk patients, who are less likely to benefit and reduce waiting times for high-risk patients who require more regular surveillance. In addition, as most adenoma patients face a lifetime of burdensome colonoscopies with its associated bowel preparation and procedural risks, targeting surveillance to high-risk patients would also likely increase compliance with recommended follow-up colonoscopy intervals, which is often poor; only 36% of patients comply with clinical guideline recommended intervals for surveillance colonoscopy in Australia (31). Moreover, with increasing incidence in CRC seen in younger age groups (32, 33), especially those under eligibility age thresholds for FOBT programs (34), and differential surveillance colonoscopy compliance based on patient insurance status (35), risk stratification holds additional benefits for particular patient groups.

The literature on risk stratification for CRC prevention primarily incorporates factors such as family history and sociodemographics (age, sex, and socioeconomic status) with some models also incorporating genetic variants associated with CRC susceptibility (36). Where surveillance colonoscopy is considered, adenoma number, size, villosity, and dysplasia grade at the most recent investigation are the more common determinants for recommending future surveillance intervals, whereas other factors including proximal or distal adenoma location, and the total adenoma burden over time are often overlooked as risk factors for future CRC.

## Incorporating Data from Multiple Prior Colonoscopies

The cumulative burden of prior colorectal adenoma has almost exclusively been omitted from risk stratification approaches for surveillance colonoscopy, often due to unavailability of data. Most research in this area has only incorporated data from the most recent colonoscopy. However, it is likely that the risk of adenoma recurrence or development of CRC is modified by prior adenoma and/or changes in adenoma characteristics over time. Therefore, risk increases are likely conditional on adenoma characteristics from multiple earlier examinations rather than just the most recent investigation.

To date, there has been little published work which has considered longitudinal colonoscopy history for risk prediction of CRC. Estimates from a relatively small study (*n* < 3,000) of Dutch patients investigated predictive ability of baseline colonoscopy on adenoma burden for up to two subsequent colonoscopies (37). The authors reported that optimizing timing of colonoscopy surveillance by incorporating multiple risk factors could result in 20% fewer surveillance colonoscopies being required annually, while maintaining the same level of effectiveness in terms of cancer detection and life-years gained (37). Three other studies have reported on rates of advanced adenoma or CRC incorporating up to two surveillance colonoscopies (38–40), although, as commented by the US Multi-Society Task Force on Colorectal Cancer (41), all have important limitations possibly resulting in selection bias. Despite these weaknesses, findings were consistent across these studies suggesting that accounting for longitudinal colonoscopy history could provide important information for CRC risk prediction. While these results are encouraging, there is currently a complete lack of findings in the literature beyond the second surveillance colonoscopy. Consequently, the extent to which adenoma burden over a patient’s life mediates future CRC risk is largely unknown.

Due to the lack of empirical data in this area, recommended intervals for follow-up colonoscopy in most national clinical guidelines, such as those in the US, UK, Australia, and Europe (15, 41–43), are almost exclusively based on results of the latest examination alone. Consequently, existing international guidelines are arguably a compromise that may not accurately define optimal intervals for repeat surveillance in patients with detected adenomas over multiple prior colonoscopies.

In Australia, clinical guidelines advocate that a risk assessment combining the results at baseline and at least one repeat surveillance examination may be a superior tool for CRC prediction than reliance on findings at the latest examination (15). However, there is no guidance provided on how to use that information other than a general statement that endoscopists should be encouraged to consider previous colonoscopy findings. The authors of the Australian Clinical Guidelines for Colonoscopy Surveillance recognize this limitation and recommend further research to determine CRC risk after a series of surveillance examinations, stratified by risk parameters of the baseline adenomas (15). This has also been highlighted as an important area in an Australian gap analysis (44).

## Opportunities in the Current Data Environment

The emergence of whole-population data linkage systems in many countries has afforded the opportunity to combine comprehensive data from a range of health service data collections for large samples over decades. Such linkage systems provide a powerful resource for conducting longitudinal research on large or even entire populations and have benefits for minimizing, if not overcoming, limitations due to sample size, selection bias, response or recall bias, loss-to-follow-up, and ascertainment of accurate health service exposure and outcome measures. The use of such data has become commonplace in health research (45), and linkage of whole-population non-consented service data for research purposes is an accepted ethical approach (46).

Data from such linkage systems could also lay the foundation for more robust risk stratification of populations, incorporating a wide range of sociodemographic, clinical, and genetic factors depending on the data available to be linked. Linkage systems, such as the Western Australian Data Linkage System (47), use widely accepted probabilistic-matching techniques and already have capacity to link decades of cancer registry, inpatient, pathology, and mortality data, combined with the ability to genealogically link patients at the individual-level to derive familial history of disease and “genetic” risk factors. Such data provide a unique platform to investigate different risk stratification models for CRC detection through colonoscopy surveillance. Moreover, due to the extensive observation periods that can be investigated, these systems provide the opportunity to incorporate data based on findings over multiple surveillance colonoscopies, which have been omitted from the literature to date but are likely an important component for precision targeting of ongoing surveillance windows. Additional linkage to National Bowel Cancer Screening Program records and large cohort studies, which may provide information on a range of health behaviors not routinely captured in administrative data such as smoking, alcohol consumption, diet, and physical activity would further enhance the ability to precisely stratify CRC risk and tailor appropriate follow-up intervals. The lack of such behavioral risk factor information, rarely captured in administrative data, is a potential limitation and arguably does not allow all risk factors to be considered in risk stratification models. However, available administrative data do allow targeting of factors most relevant to guideline-based decision making in this area. Furthermore, the approach proposed in this paper would still provide an advance on existing risk-stratification models as a result of accounting for the cumulative burden of prior colorectal adenoma which has been omitted from risk stratification approaches to CRC screening and surveillance to date.

In addition, when combined with the availability of tools such as MISCAN-Colon, a well-established microsimulation model for CRC (48, 49), evaluation of the cost-effectiveness of different risk stratification models for informing timing of ongoing follow-up colonoscopy for CRC is possible. Such work can also be tailored to jurisdictional-specific settings and precedents exist for the adaption of the MISCAN-Colon model to local settings, such as the Australian-specific variant of MISCAN-Colon (50).

## Conclusion

Whole-population data linkage systems are uniquely placed to allow robust longitudinal investigation to develop risk stratification models for CRC surveillance. Systems would require the capacity to link data collections comprising demographic, cancer registry, hospital inpatient, pathology, mortality, and genealogical factors over multiple decades at the whole-of-population level. The ability to link additional behavioral risk factor data (e.g., smoking, alcohol consumption, and dietary intake) from sources such as large cohort studies would also add value. The linking of such data collections would allow relevant risk factors to be accounted for in risk stratification models, including the incorporation of complete colonoscopy history and adenoma burden over time, which represents a potentially important modifying factor for cancer risk but is currently not included in risk modeling for recurrent adenoma of CRC.

In addition to providing greater precision with patient risk profiling, estimates can be used in cost-effectiveness analyses to determine optimal colonoscopy surveillance intervals for patients at different levels of cancer risk. This could reduce costs to the health system without a reduction in the number of CRCs that surveillance colonoscopy prevents. Such information also has capacity to support rational decisions concerning the best strategy for repeat surveillance via colonoscopy for patients at both low and high risk for CRC and reduce excessive delays for surveillance colonoscopy, especially for high-risk patients. Moreover, it creates an evidence-base for recommendations that would be immediately implementable in clinical practice with the potential to influence national colonoscopy surveillance guidelines.

## Author Contributions

DP was the lead investigator for the project to which this opinion piece relates and was responsible for concept development, undertaking the relevant literature critique and drafting the initial manuscript. IL-V provided direct health economics and colorectal cancer screening expert input and was involved (along with DP) with developing the overall concept. PO and DC provided genetic and risk stratification for cancer screening input. HE and CP provided colorectal clinical and surgical input. MB and LT provided methodological expertise. All the authors were involved with developing the manuscript and provided detailed feedback and commentary on all iterations of the draft paper.

## Conflict of Interest Statement

The authors declare that the research was conducted in the absence of any commercial or financial relationships that could be construed as a potential conflict of interest. The reviewer JC declared a shared affiliation, with no collaboration, with one of the authors MB to the handling Editor.
